# Evaluation of Insecticidal Potentials of Five Plant Extracts against the Stored Grain Pest, *Callosobruchus maculatus* (Coleoptera: Bruchidae)

**DOI:** 10.3390/insects13111047

**Published:** 2022-11-13

**Authors:** Rasheed Akbar, Imtiaz Ali Khan, Reem A. Alajmi, Ashraf Ali, Brekhna Faheem, Amjad Usman, Ashraf M. Ahmed, Mohamed El-Shazly, Abid Farid, John P. Giesy, Mourad A. M. Aboul-Soud

**Affiliations:** 1 Department of Entomology, Faculty of Basic and Applied Sciences, The University of Haripur, Haripur 22062, Pakistan; 2 Department of Entomology, The University of Agriculture, Peshawar 25130, Pakistan; 3 Department of Zoology, College of Science King Saud University, Riyadh 11451, Saudi Arabia; 4 Department of Chemistry, Faculty of Natural Sciences, University of Haripur, Haripur 22062, Pakistan; 5 Department of Zoology, Abdul Wali Khan University, Mardan 23200, Pakistan; 6 Pharmacognosy Department, Faculty of Pharmacy, Ain-Shams University, Organization of African Unity Street, Abassia, Cairo 11566, Egypt; 7 Pharmaceutical Biology Department, Faculty of Pharmacy and Biotechnology, The German University in Cairo, New Cairo 12613, Egypt; 8 Toxicology Centre, University of Saskatchewan, Saskatoon, SK S7N 5B3, Canada; 9 Department of Veterinary Biomedical Sciences, University of Saskatchewan, Saskatoon, SK S7N 5B4, Canada; 10 Department of Integrative Biology, Michigan State University, East Lansing, MI 48824, USA; 11 Department of Environmental Sciences, Baylor University, Waco, TX 76706, USA; 12 Department of Clinical Laboratory Sciences, College of Applied Medical Sciences, King Saud University, Riyadh 11433, Saudi Arabia

**Keywords:** entomotoxicity, stored product pests, plant extracts, phytochemicals, bruchid beetle, bio-pesticides

## Abstract

**Simple Summary:**

Conventional pesticides are synthetic chemicals that are toxic, have hazardous effects on living organisms and may be carcinogenic. Pests can be controlled in an alternative way using less hazardous and more ecofriendly methods, such as using bio-pesticides. Plants based pesticides are chemicals isolated from various plants which could be used to control pests in a non-toxic mechanism. Several plants have certain bioactive compounds which could be used as alternatives to hazardous synthetic pesticides for pest control. Some beetles could damage stored grains and thus causes huge economic losses. For example, *Callosbruchus maculatus* is a stored grain pest which causes more than 90% damage to sored grains in a few months. Here, in this work, the insecticidal potentials of six plants, including Melia azedarach, Nicotiana rustica, Azadirachta indica, Nicotiana tabacum and Thuja orientalis, were investigated against *C. maculatus*. Crude extracts of these plants at different concentration were checked against *C. maculatus* and mortality was observed. Maximum mortality was caused by N. tabacum and N. rustica (100%) followed by A. indica (82%), whereas minimum mortality was observed in T. orientalis (64%) at 2.5%. The results of this study revealed that the extracts of these plants could be used for the control of stored grain pests.

**Abstract:**

Plant based insecticides are considered among the most economic and ecofriendly chemicals for the protection of plants and stored grains. The cowpea weevil (*Callosbruchus maculatus*) causes more than 90% damage to sored grains in three to six months. The current study investigates insecticidal potentials of five selected botanicals: *Melia azedarach, Nicotiana rustica, Azadirachta indica*, *Nicotiana tabacum* and *Thuja orientalis.* They are explored at six different concentrations (0.5, 1.0, 1.5, 2.0, 2.5 and 3.0%) against *C. maculatus* and compared to effects of distilled water which is used as a control. Toxicities of 3%(V/V) extracts of *N. tabacum, N. rustica, A. indica* and *T. orientalis* against *C. maculatus* were 100%, 86.11%, 80.56% and 72.22%, respectively. Maximum mortality was caused by *N. tabacum* and *N. rustica* (100%), followed by *A. indica* (82%), whereas minimum mortality was observed in *T. orientalis* (64%) at 2.5%. Several phytochemicals, alkaloids, saponins, diterphenes, phytosterol, flavonoids and phenols were identified in *N. tabacum* and *N. rustica,* while few were present in *A. indica.* Phytosterol was present in greatest abundance. Saponins were only detected in aqueous extracts of *N. rustica* and *N. tabacum.* Taken together, these results indicate the utility of *N. tabacum*, *N. rustica* and *A. indica* as potential botanicals to control pest beetle and cowpea weevil.

## 1. Introduction

The demand for food increases with an ever-growing population. Thus, it is necessary to protect crops and stored grains from pests. The multivoltine pest, cowpea weevil *Callosobruchus maculatus* Fab. (Coleoptera: Bruchidae), causes significant damage to stored pulses [1]. It has been reported that *C. maculatus* alone can cause as much as 90% damage during the three to six months of storage [2]. Due to their potencies to cause lethality in most life stages of a range of pests, synthetic pesticides are frequently used to protect both crop plants and stored grains [3]. Synthetic pesticides can adversely affect non-target organisms, including humans, accumulate in the environment, pollute soil and ground water. Some of the synthetic pesticides are also carcinogenic [4]. The overuse of synthetic pesticides for insect control poses risks to wildlife and even humans [5]. Toxic potency of synthetic pesticides and their potential effects have stirred interest from the public and regulatory agencies in alternative options for pest management [6].

Until the relatively recent development of synthetic pesticides and their widespread application since the 1940s, phytochemicals have long been successfully used to manage pests in crops [7]. Extracts of plants and other secondary metabolites of plants, microorganisms and enzymes are becoming increasingly popular as alternatives to synthetic pesticides [8]. Bio-insecticides can be very effective, selective and have little potential for developing resistance to target pests, as well as having minimal effects on non-target organisms [9].

Several plant extracts have been used to control various stored insect pests. Essential oils of some aromatic plants have been recognized to have cytotoxic, antioxidant, antifungal, insecticidal and antibacterial properties [10]. The aqueous extract of neem kernel has been used to protect crops from infestation with pests [11,12]. The species of the deciduous tree in the mahogany family, Meliaceae, commonly known as the chinaberry tree, pride of India, bead-tree, Cape lilac, syringa berry tree, Persian lilac, Indian lilac and white cedar (*Melia azedarach*) have insecticidal properties against several pest species [13]. The tobacco plants *Nicotiana. tabacum* and *Nicotiana. rustica* are also able to control several insect pests. Nicotine present in *N. tabacum* and *N. rustica* cause uncontrolled nerve firing and masking acetylcholine in insects, which results in death [13]. The present study was conducted to evaluate insecticidal potencies of extracts of five plants against *C. maculatus.* The toxic potencies of the five botanicals, *M. azedarach, N. rustica, A. indica*, *N. tabacum* and *T. orientalis,* against the cowpea weevil, *C. maculatus*, are investigated.

## 2. Materials and Methods

### 2.1. Collection of Insects

Cowpea weevil, *Callosobruchus maculatus*, were collected from various regions of the Swabi and Haripur districts of the Khyber Pakhtunkhwa province in Pakistan. *C. maculatus* were taken to the entomological laboratory and identified according to previous reports [14]. Adults of *C. maculatus* were chosen to initiate the culture at the entomological laboratory under laboratory conditions of 27.5 °C, 60 ± 5% RH and a 12L:12D photoperiod on the entire mung bean (*Vigna radiate* L.), which is known to be an ideal host in plastic jars (10 × 12 cm) enclosed in muslin fabric [15].

### 2.2. Plants Collection

Leaves and fruits of the specified selected plants were collected from various areas in the Swabi and Haripur districts of the Khyber Pakthunkhwa in Pakistan, according to previously described procedures [16] (Table 1).

### 2.3. Phytochemical Screening

Extracts of selected plants were screened for the presence of various bio-active compounds, such as alkaloids, phenols, phytosterol, terpenes and flavonoids. The detection of these compounds was carried out using standard tests, as reported in literature.

#### 2.3.1. Maceration

Coarsely powdered plant material was extracted in a container of methanol and ethyl acetate and hexane) and agitated for a defined period.

#### 2.3.2. Detection of Alkaloids, Phenols, Phytosterol, Terpenes and Flavonoids in Plant Extract

A few drops of iodine and two to three drops of potassium iodide were dissolved individually in diluted hydrochloric acid (1.5%). One milliliter of this reagent was added to plant extracts and stirred for five minutes. Dark reddish precipitates were observed in the samples, which indicates the presence of alkaloids in plants extracts [17,18]. For the screening of phenol, three to four drops of ferric chloride solution were added to plant aqueous extracts. The formation of a bluish black color indicates the presence of phenols in the extract [18]. Similarly, for the detection of phytosterol, extracts of the selected plants were cured with chloroform and then filtered. Few drops of concentrated sulfuric acid were added into the filtered extract and allowed to be undisturbed for a few minutes. The formation of a golden yellow color indicates the presence of phytosterol [19]. For the determination of diterpenes in the selected plants extracts, three to four drops of copper acetate solution were added into the aqueous extracts of the selected plants. The formation of emerald green color indicates the presence of diterpenes [20]. Flavonoids were detected in plants extracts by adding two to three drops of lead acetate solutions to the plant aqueous extracts. A bright yellow color appeared, which indicates the presence of flavonoids in the sample. The color disappeared when few drops of dilute acid was added [21].

### 2.4. Residual Toxicity

Residual toxicity of extracts was determined according to previously described methods (22). In this study, five plant extracts and six different concentrations (Table 2) were tested, following the CRD design with (6 × 5) factorial arrangement with four replicates. Sterilized test sample of mung bean (20 g) were treated with plant extracts (Table 2) separately, then air-dried for 30 min. Treated legumes were then put in plastic petri dishes (12 cm diam.). Five pairs of newly emerged adults were released in each petri dish and covered with muslin cloths to prevent the escapes of beetles from the testing arena. Mortality of *C. maculatus* in each petri dish was assessed after 24, 48, 72, 168 and 336 h of exposure. Dead beetles from each petri dish were removed and counted. Corrected mortality (23) for each treatment was calculated as per the following formula (Equation (1)).

(1) $$Corrected\;\%\;Mortality=\frac{\%\;mortality\;in\;treatment-\%\;mortality\;in\;control}{100-\%\;mortality\;in\;control}\times 100$$

### 2.5. Topical Toxicity

Topical toxicity of the plant extracts (Table 2) was also investigated against *C. maculatus* adult beetles. The same experimental design and treatment in Section 2.4 were applied here. The concentrations of plant extracts (Table 2) were applied through pipet onto the thoracic segment of *C. maculates,* then wrapped in aluminum foil and stored in the refrigerator for five minutes before being treated. All treated *C. maculatus* adults were put in plastic petri plates with the help of a camel hairbrush. After exposure for 24, 48, 72, 168 and 336 h, the number of dead beetles was counted and the percentage mortality (%) for each concentration was calculated.

### 2.6. Statistical Analysis

While normality was checked using the Shapiro–Wilks test, the assumption of homogeneity of variance was evaluated using Levene’s test. The Tukey HSD analysis was applied at 5% probability of Type I error (α) to separate the means of the obtained data from recent research using Analysis of Variance (ANOVA). Statistical analyses were carried out using STATISTIX 8.1 (24). The mortality percentages were corrected using Abbott’s formula. Thereafter, the Log-Probit model analysis was applied to percentage mortality of the adult *C. maculatus* to determine the 50% and 90% lethal concentrations (LC50/LC90) (25). The Analysis of Variance and the Probit analyses were done using the Statistical Package for the Social Sciences (SPSS) version 20.

## 3. Results

Several phytochemicals, including alkaloids, saponins, di-terphenes, phyto-sterol, flavonoids and phenols, were identified in *N. tabacum* and *N. rustica,* while few were present in *A. indica.* Phytosterol was present in greatest abundance. Saponins were detected only in aqueous extracts of *N. rustica* and *N. tabacum* (Table 3). 

**Figure 1:**
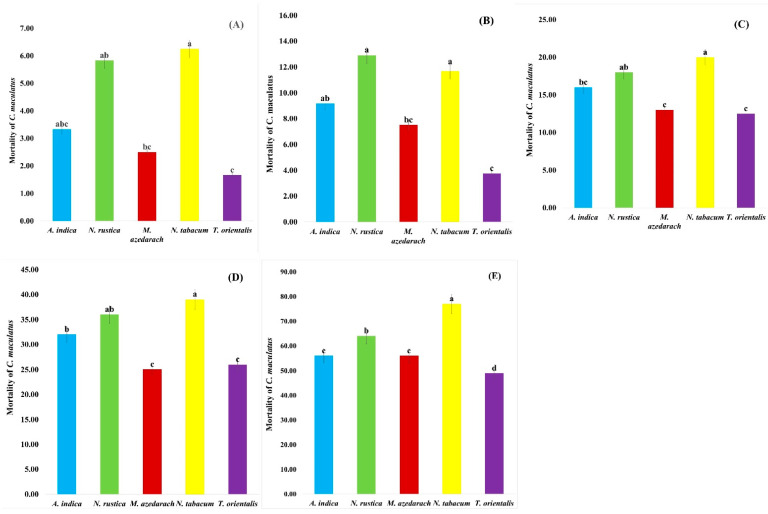
Mean percent Mortality of *C. maculatus* treated with plant extracts after (**A**) 24 h, (**B**) 48 h, (**C**) 72 h, (**D**) 168 h and (**E**) 336 h exposure (Residual effect). (a, b, c, d put over the bars indicating that different letters are significantly different from each other at 0.5% level of significance).

**Figure 2:**
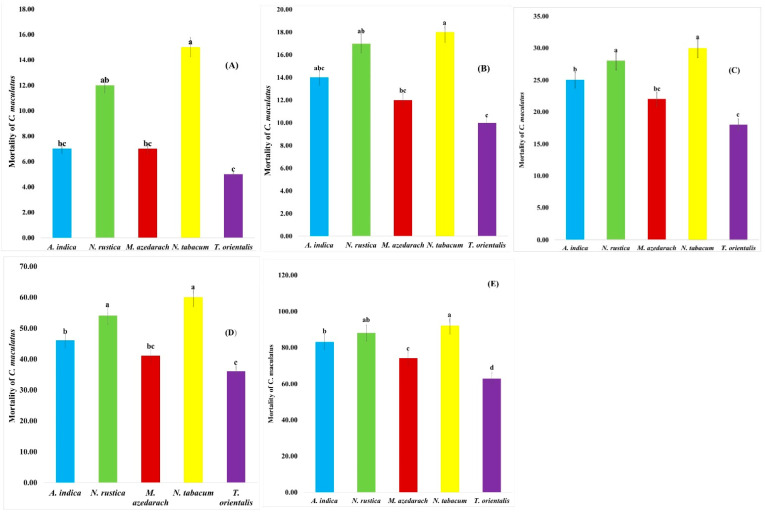
Mean percent Mortality of *C. maculatus* treated with plant extracts after (**A**) 24 h, (**B**) 48 h, (**C**) 72 h, (**D**) 168 h and (**E**) 336 h exposure (Topical effect). (a, b, c, d put over the bars indicating that different letters are significantly different from each other at 0.5% level of significance).

The insecticidal activity of plant aqueous extracts was tested against *C. maculatus* to six concentrations (0.5, 1.0, 1.5, 2.0, 2.5, and 3.0%). Data pertaining to insecticidal activity of the selected plant extracts both in residual (Figure 1 and Table 4) and direct (Figure 2 and Table 5 forms are represented.

Among the plant species extracts, the highest residual mortalities of *C. maculatus* was observed with *N. tabacum* with 6.25%, followed by *N. rustica* with 5.83% and *A. indica* with 3.33%. Lowest mortalities were observed with *T. orientalis*, i.e., 1.66%, followed by *M. azedarach*, i.e., 2.50% (df = 4, F = 10.48, *p* = 0.000), after 24 h of exposure period (Figure 1A). The LC50 values towards *C. maculatus* among the plant extracts were 24.73 for *N. tabacum*, followed by 29.73 for *M. azedarach*. The extract of *T. orientalis* with an LC50 of 54.85 was the least efficacious of the extracts (Table 4).

The exposure time was further increased to 48 h to check the effect of these selected five plants extracts on the mortality of pests. Figure 1B shows the effects of the six different concentrations of five different plant species crudely extracted after the 48-h exposure period. Crude extracts showed significant mortality on *C. maculatus*. Maximum mortalities of 12.91% were recorded with *N. rustica*, followed by *N. tabacum* with 11.67% and *A. indica* with 9.16%, while minimum mortalities to *C. maculatus* were recorded with *T. orientalis* with 3.75%, followed by *M. azedarach* with 7.50% (df = 4, F = 06.46, *p* = 0.0001). The LC50 values, based on mortality of *C. maculatus,* were 20.75 for *N. tabacum,* followed by 24.20 for *M. azedarach* (Table 4). *T. orientalis,* which exhibited values for mortality of 35.50, were the least efficacious.

Extracts of the five different tested plant species had significantly impacted the mortality of *C. maculatus* after the 72-h exposure period (Figure 1C). *C. maculatus* mortalities were higher at *N. tabacum* with 20.00%, followed by *N. rustica* with 18.00% and *A. indica* with 16.00%. Lower mortalities of 12.50% were seen with *T. orientalis*, followed by *M. azedarach* with 13.00% (df = 30, F = 07.11, *p* = 0.0001). The LC50 value for *N. tabacum* against *C. maculatus* was 17.53, followed by *N. rustica* with 24.39 and *T. orientalis* with 29.01 (Table 4).

Results represented in Figure 1D indicate significant mortality rates of the five different plant species crudely extracted for six different concentrations after 168 h of exposure to *C. maculatus*. The mortality rates of *C. maculatus* increase as the exposure time increases. *C. maculatus* mortalities were higher with *N. tabacum* with 39.00%, followed by *N. rustica* with 36.00% and *A. indica* with 32.00%. Lower mortalities of 25.00% were recorded with *T. orientalis,* followed by *M. azedarach* with 26.00% (df = 4, F = 20.30, *p* = 0.0000). From Table 4, it was clear that the LC50 of *N. tabacum* is 4.75, followed by *N. rustica* with 5.44 and *A. indica* with 6.32, whereas *T*. *orientalis* and *M. azedarach* have LC50 values of 8.39 and 9.15, respectively.

Finally, results presented in Figure 1E revealed significant mortality rates of five different plants species crude extracts of six different concentrations after fourteen days of exposure to *C. maculatus*. The highest mortalities of *C. maculatus* were noted with *N. tabacum* with 77.00%, followed by *N. rustica* with 56.00%. *A. indica* and *M. azedarach* exhibited the same mortalities of 56.00%, while the lowest mortalities of 49.00% were seen with *T. orientalis* (df = 4, F = 33.80, *p* = 0.0000). In case of LC50 values from Table 4, it was clear that *N. tabacum* revealed 0.92, followed by *N. rustica* with 1.34 and *A. indica* with 1.72, whereas *T. orientalis* and *M. azedarach* resulted in 2.55 and 1.86, respectively. The mean percent (±SE) residual mortality of *C. maculatus* after various time intervals is given in the supplementary file (Tables S1–S5) and the mean percent (±SE) topical mortality of *C. maculatus* after various time intervals is given in the supplementary file (Tables S6–S10).

Among the plant species extracts, the highest mortalities of *C. maculatus* were observed with *N. tabacum* of 15.00%, followed by *N. rustica* with 12.00%. The lowest mortality was observed with *T. orientalis* with 7.00%, followed by *A. indica* and *M. azedarach,* both with 7.00% (df = 4, F= 10.48, *p* = 0.00) after the 24-h exposure period (Figure 2A). In case of LC50, *N. tabacum* had 28.84, followed by *N. rustica* and *M. azedarach* with 36.33, were most effective. *T. orientalis* (51.87) was least effective against *C. maculatus* (Table 5).

The exposure time was further increased to 48 h to check the effect of these selected five plants extracts on the mortality of pests. Figure 2B shows the effects of six different concentration of five different plant species crudely extracted after the 48-h exposure period. Crude extracts showed significant mortality on *C. maculatus*. A maximum mortality of 18.00% was recorded with *N. tabacum*, followed by *N. rustica* with 17.00% and *A. indica* with 14.00%. A minimum mortality of 10.00% to *C. maculatus* was recorded with *T. orientalis,* followed by *M. azedarach* with 14.00% (df = 4, F = 06.46, *p* = 0.0001). From Table 5, it was clear that among the plant species *N. tabacum* (16.23), followed by *N. rustica* (28.59) and *M. azedarach* (32.31), gave promising results in killing 50% of the tested population of *C. maculatus*. *T. orientalis*’s (40.86) mortality showed the lowest effectiveness.

Extracts of five different tested plant species had significantly impacted the mortality of *C. maculatus* after the 72-h exposure period (Figure 2C). *C. maculatus* mortalities were higher at *N. tabacum* with 30.00%, followed by *N. rustica* with 28.00%, *A. indica* with 25.00% and *M. azedarach* with 22.00%. Lower mortalities of 18.00% were seen with *T. orientalis* (df = 30, F = 07.11, *p* = 0.0001). In case of LC50, *N. tabacum* (7.73), followed by *N. rustica* (10.21) and *A. indica* (13.08), were most effective, while *T. orientalis* (44.99) was found least effective against *C. maculatus*, as shown in Table 5.

Results represented in Figure 2D indicate significant mortality rates of five different plant species crudely extracted at six different concentrations after a 168-h exposure period on *C. maculatus*. The mortality rates of *C. maculatus* increases as the exposure time increases. *C. maculatus* mortalities were higher with *N. tabacum* by 60.00%, followed by *N. rustica* at 54.00%, *A. indica* at 46.00% and *M. azedarach* at 41.00%. A lower mortality of 36.00% was recorded with T. orientalis (df = 4, F = 20.30, *p* = 0.0000). In case of LC50, *N. tabacum* at 1.66, followed by *N. rustica* at 1.94 and *A. indica* at 2.73, were the most effective, while *T. orientalis* at 4.25 and *M. azedarach* at 3.24 were found the least effective against *C. maculatus*, as shown in Table 5.

Finally, results presented in Figure 2E revealed significant mortality rates of five different plants species crudely extracted at six different concentrations after fourteen days of exposure on *C. maculatus*. The highest mortalities of *C. maculatus* were noted with *N. tabacum* at 92.00%, followed by *N. rustica* at 88.00% and *A. indica* at 83.00%, while the lowest mortalities were seen at 63.00% with *T. orientalis* and 74.00% with *M. azedarach* (df = 4, F = 33.80, *p* = 0.0000). Among the plant extracts, *N. tabacum* at 0.19, followed by *N. rustica* at 0.20, were more promising in killing 50% of the tested population of *C. maculatus*. *T. orientalis* at 0.31 mortality showed the lowest effectiveness, as shown in Table 5.

## 4. Discussion

### 4.1. Mortality of C. maculatus Exposed to Each of Five Plants

Crude extracts of *A. indica, N. tabacum. N. rustica, M. azedarach* and *T. orientalis* were mixed with 20 g mung bean at the rate of 0.5%, 1%. 1.5, 2%. 2.5% and 3%, respectively. At all the concentrations tested, crude extracts of *N. tabacum, N. rustica, A. indica* and *M. azedarach* showed more residual toxicity against *C. maculatus*. Our findings are similar to the findings of a past study [22] suggesting that crude extracts of *N. tabacum* had the highest residual toxicity against *Tribolium castaneum*, i.e., 89% at a 3% concentration. Botanical insecticides contain a range of bioactive compounds that have potential to cause adverse effects on organisms that either consume them or are exposed to them. In particular, plants have co-evolved with insects that eat them, so they have developed defense mechanisms, including production of compounds that disrupt normal physiology and behavior of insects, thus, affecting eating, mating, mortalities and oviposition [23]. Secondary metabolites, *N. tabacum* and *N. rustica*, including alkaliods, flavoniods, saponins and di-tarphene can repel or kill insects. Alkaloids, including nicotine, act as stomach poison in insects. Tobacco leaves contain physiologically active chemicals that paralyze insects by acting on their central nervous system. When they feed on plants containing these compounds insects ultimately die [24]. It has recently been observed that when relatively large amounts of *A. indica* was consumed in the diet of *C. maculatus*, significant mortalities were observed [25]. The major mechanism of action of azadirichtin has been reported to limit the release of neurosecretory material from the corpora cardiaca, resulting in a slower turnover rate, as well as altering the prothoracicotropic hormone (PTTH) via brain neurosecretory cells [26]. The results of this study are consistent with those reported previously suggesting that pulses sprayed with crude extracts of *M. azedarach* were well-preserved for up to six months without evidence of infestation [27]. Results of this study revealed insecticidal potency via topical application through consumption in the diet of *C. maculatus* without treating any mung bean seeds. Among the plants tested, *N. tabacum*, followed by *N. rustica, A. indica* and *M. azedarach*, exhibited significantly maximum accumulative mortality, whereas *T. orientalis* caused minimum mortality. The mechanism of entrance of the active components into the target location in insects can also be attributed to variations in potencies to cause mortality of the extracts. Biocidal ingredients of extracts are thought to enter the insect through the integument [28]. Specifically, toxins have been reported to gain access to the target areas through the lipophilic and hydrophilic cuticle, where they cause a variety of effects through multiple mechanisms. The hydrophilic-hydrophobic structure of the cuticle has been reported to influence penetration of pesticides which also controls their efficacy [29]. Chemical properties of active principals and polarity influence passage of bioactive compounds, like insecticides through the cuticle. The cuticle’s outermost lipophilic phase promotes nonpolar molecular mobility. As a result, only toxins in extracts with a favorable polarity and chemical composition were able to penetrate to an internal site of toxic action, which could initiate molecular responses that result in lethality. A dose-response relationship was observed between the concentration of a given crude plant extract and the corresponding percent mortality, which is consistent with the results reported previously [30].

### 4.2. Mortality of Selected Plants Extracts against C. maculatus

The harmful effects of the compounds in extracts of the studied plants observed in this study might have contributed to the mortality of *C. maculatus,* which is in close agreement with other similar works reported in literature [31]. Even though all of the plants exhibited promise as insecticides, their toxic potencies against *C. maculatus* differed, most likely due to differences in phytochemical composition. Currently *N. tabacum* exhibited the greatest mortality of *C. maculatus* at concentrations of 3% [32]. This also confirms the same results that *N. tabacum* exhibits high mortality of pulse beetle at 3% concentration. As suggested previously, secondary metabolites contained in these plants might be responsible incapacity of adult *C. maculatus* [33].

### 4.3. Phytochemical Analysis Five Plants Extracts

*Nicotiana tabacum* contains relatively great concentrations of alkaloids, phenolic compounds, flavonoid, tannins, saponins, terpenoids, various proteins and carbohydrates. This result is consistent with previous reports of constituents in extracts of this plant. Nicotine, the active ingredient of *N. tabacum*, has been documented to have contact, stomach, and respiratory poisoning effects [33]. Saponins, alkaloids, flavonoids, tannins and cyanogenic glucosides are all found in crude extracts of *A. indica*. The natural phytochemicals from plants have a potential of being eco-friendly and replace synthetic pesticides for insect pests [34].

## Figures and Tables

**Table 1:** List of plant species and plant parts tested against *C. maculatus* during 2021.

Sr. No.	Common Name	Botanical Name	Family	Part Used
1.	White Patta	*Nicotiana rustica*	Solanaceae	leaf
2.	Virginia tobacco	*Nicotiana tabacum*	Solanaceae	leaf
3.	Chinese arborvitae	*Thuja orientalis*	Cupressaceae	Fruit
4.	Neem	*Azadirachta indica*	Meliaceae	Seed
5.	Bakion	*Melia azadarech*	Meliaceae	Fruit

**Table 2:** Plant extract and their concentrations used against *C. maculatus* during 2021.

Sr. No.	Common Name	Botanical Name	Concentration Used
1.	White Patta	*Nicotiana rustica*	0.5, 1, 1.5, 2, 2.5 and 3%
2.	Virginia tobacco	*Nicotiana tabacum*	0.5, 1, 1.5, 2, 2.5 and 3%
3.	Chinese arborvitae	*Thuja orientalis*	0.5, 1, 1.5, 2, 2.5 and 3%
4.	Neem	*Azadirachta indica*	0.5, 1, 1.5, 2, 2.5 and 3%
5.	Bakion	*Melia azadarech*	0.5, 1, 1.5, 2, 2.5 and 3%

**Table 3:** Composition of phytochemicals in aqueous extracts of five selected plants species.

	Phytochemical Constituents of Five Plant Species					
Plant Species	Alkaloids	Flavonoids	Saponins	Di-Terpenes	Phyto-Sterol	Phenols
*T. orientalis*	Low	low	low	low	Low	low
*M. azedarach*	low	low	low	low	moderate	moderate
*N. rustica*	low	low	not present	low	Low	low
*A. indica*	High	high	moderate	moderate	High	high
*N. tabacum*	moderate	moderate	moderate	high	moderate	high

**Table 4:** Mortality of *C. maculatus* exposed to aqueous extracts for 24, 48, 72, 168 and 336 h.

Time Hours	Plant Species	N*	LC_50_ 95% LC	X2	P	Slope + SE
24	*A. indica*	240	38.44 (15.77–740.47)	0.85	0.93	0.96 ± 0.26
	*N. rustica*	240	37.19 (16.02–495.66)	1.59	0.80	1.07 ± 0.28
	*M. azedarach*	240	29.73 (16.01–359.42)	2.28	0.68	1.44 ± 0.37
	*N. tabacum*	240	24.73 (18.12–1308.62)	0.69	0.95	1.05 ± 0.29
	*T. orientalis*	240	54.85 (18.42–1892.92)	1.37	0.84	1.50 ± 0.53
48	*A. indica*	240	32.24 (12.10–366.33)	1.73	0.78	2.36 ± 0.75
	*N. rustica*	240	30.27 (14.92–224.42)	0.82	0.58	0.63 ± 0.37
	*M. azedarach*	240	24.20 (9.91–335.57)	1.62	0.80	3.43 ± 1.40
	*N. tabacum*	240	20.75 (23.91–6190.66)	0.99	0.91	1.22 ± 0.37
	*T. orientalis*	240	35.50 (13.60–7305.06)	1.76	0.78	2.13 ± 0.79
72	*A. indica*	240	28.36 (13.71–217.70)	1.17	0.88	1.06 ± 0.26
	*N. rustica*	240	24.39 (12.49–148.61)	0.40	0.98	1.06 ± 0.26
	*M. azedarach*	240	29.57 (14.34–220.30)	0.49	0.97	1.16 ± 0.28
	*N. tabacum*	240	17.53 (10.27–62.95)	1.39	0.84	1.12 ± 0.24
	*T. orientalis*	240	29.01 (14.35–194.43)	2.37	0.66	1.22 ± 0.29
168	*A. indica*	240	6.32 (4.89–9.95)	1.75	0.78	1.17 ± 0.21
	*N. rustica*	240	5.44 (4.23–8.48)	3.06	0.54	1.06 ± 0.20
	*M. azedarach*	240	9.15 (5.40–101.82)	7.04	0.13	1.18 ± 0.22
	*N. tabacum*	240	4.75 (3.74–7.01)	3.16	0.53	1.03 ± 0.20
	*T. orientalis*	240	8.39 (6.20–14.91)	1.76	0.77	1.23 ± 0.22
336	*A. indica*	240	1.86 (1.43–2.24)	4.94	0.29	1.40 ± 0.20
	*N. rustica*	240	1.34 (0.93–1.69)	4.83	0.30	1.39 ± 0.20
	*M. azedarach*	240	1.72 (1.21–2.15)	6.58	0.16	1.19 ± 0.20
	*N. tabacum*	240	0.92 (0.04–1.61)	17.36	0.00	1.79 ± 0.23
	*T. orientalis*	240	2.55 (2.10–3.02)	3.94	0.41	1.41 ± 0.20

N* = number of insects used; LC Lethal concentration are indicated with 95% confidence limit (CL,). LC50 of plant extract g/mL.

**Table 5:** Toxicological effect (Direct) of plant extracts against *C. maculatus* after 24, 48, 72, 168 and 336 h’ exposure period.

Time	Plant Species	N*	LC-50 95% LC	X2	P	Slope + SE
24	*A. indica*	240	44.55 (17.88–987.72)	1.40	0.57	0.55 ± 0.16
	*N. rustica*	240	36.33 (15.83–462.57)	0.23	0.99	0.47 ± 0.12
	*M. azedarach*	240	36.33 ((15.83–462.57)	0.23	0.99	0.47 ± 0.12
	*N. tabacum*	240	28.84 (13.47–278.34)	0.05	1.00	0.42 ± 0.11
	*T. orientalis*	240	51.87(19.25–1985.44)	2.92	0.84	0.54 ± 0.15
48	*A. indica*	240	29.78 (13.83–287.89)	0.89	0.99	0.40 ± 0.11
	*N. rustica*	240	28.59 (12.92–362.31)	0.04	1.00	0.38 ± 0.10
	*M. azedarach*	240	32.31 (14.06–474.14)	0.32	0.98	0.39 ± 0.10
	*N. tabacum*	240	16.23 (9.38–65.90)	0.31	0.98	0.42 ± 0.10
	*T. orientalis*	240	40.86 (16.18–921.48)	0.825	0.92	0.39 ± 0.11
72	*A. indica*	240	13.08 (6.60–742.10)	1.43	0.83	0.92 ± 0.21
	*N. rustica*	240	10.21 (5.71–176.58)	2.43	0.65	0.94 ± 0.21
	*M. azedarach*	240	15.13 (7.23–1536.29)	0.91	0.92	0.93 ± 0.22
	*N. tabacum*	240	7.73 (4.92–37.88)	2.37	0.66	1.05 ± 0.21
	*T. orientalis*	240	23.97 (11.25–287.51)	2.00	0.73	0.68 ± 0.23
168	*A. indica*	240	2.73 (1.92–3.68)	2.12	0.71	1.32 ± 0.32
	*N. rustica*	240	1.94 (1.17–2.57)	7.90	0.09	1.35 ± 0.32
	*M. azedarach*	240	3.24 2.03–5.76)	4.33	0.36	0.94 ± 0.31
	*N. tabacum*	240	1.66 (1.06–2.16)	11.09	0.02	1.64 ± 0.32
	*T. orientalis*	240	4.25 (2.93–9.40)	3.84	0.42	1.00 ± 0.32
336	*A. indica*	240	0.31 (0.00–0.082)	7.88	0.09	1.29 ± 0.25
	*N. rustica*	240	0.32 (0.00–0.89)	12.85	0.01	1.55 ± 0.28
	*M. azedarach*	240	0.20 (0.00–0.54)	1.46	0.83	0.79 ± 0.22
	*N. tabacum*	240	0.19 (0.001–0.56)	8.82	0.06	1.43 ± 0.31
	*T. orientalis*	240	0.55 (20.01–9.40)	3.89	0.42	0.61 ± 0.20

N*= number of insects used; LC Lethal concentration are indicated with 95% confidence limit (CL,). LC50 of plant extract gm/mL.

## Data Availability

All data pertinent to this work are presented in the paper. Any requests should be directed to the corresponding author.

## Supplementary Materials

The following supporting information can be downloaded at: https://www.mdpi.com/article/10.3390/insects13111047/s1, Table S1: Mean percent (±SE) residual mortality of *C. maculatus* after 24 h exposure period treated with six different concentrations of plant crude-extracts of five plant species under laboratory conditions during 2018–2019. Table S2: Mean percent (±SE) residual mortality of *C. maculatus* after 48 h exposure period treated with six different concentrations of plant crude-extracts of five plant species under laboratory conditions during 2018–2019. Table S3: Mean percent (±SE) residual mortality of *C. maculatus* after 72 h exposure period treated with six different concentrations of plant crude-extracts of five plant species under laboratory conditions during 2018–2019. Table S4: Mean percent (±SE) residual mortality of *C. maculatus* after 168 h exposure period treated with six different concentrations of plant crude-extracts of five plant species under laboratory conditions during 2018–2019. Table S5: Mean percent (±SE) residual mortality of *C. maculatus* after 336 h exposure period treated with six different concentrations of plant crude-extracts of five botanicals under laboratory conditions during 2018–2019. Table S6: Mean percent (±SE) topical mortality of *C. maculatus* after 24 h exposure period treated with six different concentrations of plant crude-extracts of five plant species under laboratory conditions during 2018–2019. Table S7: Mean percent (±SE) topical mortality of *C. maculatus* after 48 h exposure period treated with six different concentrations of plant crude-extracts of five plant species under laboratory conditions during 2018–2019. Table S8: Mean percent (±SE) topical mortality of *C. maculatus* after 72 h exposure period treated with six different concentrations of plant crude-extracts of five plant species under laboratory conditions during 2018–2019. Table S9: Mean percent (±SE) topical mortality of *C. maculatus* after 168 h exposure period treated with six different concentrations of plant crude-extracts of five plant species under laboratory conditions during 2018–2019. Table S10: Mean percent (±SE) topical mortality of *C. maculatus* after 336 h exposure period treated with six different concentrations of plant crude-extracts of five plant species under laboratory conditions during 2018–2019.
