# Supplementary material for: Evaluation of Insecticidal Potentials of Five Plant Extracts against the Stored Grain Pest, Callosobruchus maculatus (Coleoptera: Bruchidae)
Source: Insects. 2022 Nov 13;13(11):1047. doi: 10.3390/insects13111047 (PMC9696586; doi:10.3390/insects13111047)
Supplement: Supplementary file 1 [file insects-13-01047-s001.zip › insects-1944300-supplementary.pdf]

ANOVA Tables

Table S1: Mean percent (± SE) residual mortality of *C. maculatus* after 24 h exposure period treated with six different concentrations of plant crude-extracts of five plant species under laboratory conditions during 2018-2019.

**Source**	**DF**	**SS**	**MS**	**F**	**P**
Replications	3	102.50	34.167		
Plants	4	396.67	99.167	5.49	0.0005
Concentrations	5	724.17	144.83	8.01	0.0000
Plants*concentrations	20	63.33	3.167	0.18	1.0000
Error	87	1572.50	18.075		
Total	119	2859.17			

Grand Mean 3.9167 CV 8.55

Table S2: Mean percent (± SE) residual mortality of *C. maculatus* after 48 h exposure period treated with six different concentrations of plant crude-extracts of five plant species under laboratory conditions during 2018-2019.

**Source**	**DF**	**SS**	**MS**	**F**	**P**
Replications	3	166.67	55.556		
Plants	4	1255.00	313.750	13.10	0.0000
Concentrations	5	2160.00	432.000	18.04	0.0000
Plants*concentrations	20	215.00	10.750	0.45	0.9777
Error	87	2083.33	23.946		
Total	119	5880.00			

Grand Mean 9.0000 CV 13.37

Table S3: Mean percent (± SE) residual mortality of *C. maculatus* after 72 h exposure period treated with six different concentrations of plant crude-extracts of five plant species under laboratory conditions during 2018-2019.

**Source**	**DF**	**SS**	**MS**	**F**	**P**
Replications	3	89.17	29.72		
Plants	4	986.67	246.67	10.29	0.0000
Concentration	5	5204.17	1040.83	43.41	0.0000
Plants *concentration	20	133.33	6.67	0.28	0.9990
Error	87	2085.83	23.98		
Total	119	8499.17			

Grand Mean 15.917 CV 16.76

Table S4 Mean percent (± SE) residual mortality of *C. maculatus* after 168 h exposure period treated with six different concentrations of plant crude-extracts of five plant species under laboratory conditions during 2018-2019.

**Source**	**DF**	**SS**	**MS**	**F**	**P**
Replications	3	110.3	36.76		
Plants	4	3414.1	853.53	18.18	0.0000
Concentrations	5	15928.5	3185.71	67.86	0.0000
Plants*concentrations	20	196.6	9.83	0.21	0.9999
Error	87	4084.5	46.95		
Total	119	23734.0			

Grand Mean 31.842 CV 09.52

Table S5: Mean percent (± SE) residual mortality of *C. maculatus* after 336 h exposure period treated with six different concentrations of plant crude-extracts of five botanicals under laboratory conditions during 2018-2019.

**Source**	**DF**	**SS**	**MS**	**F**	**P**
Replications	3	345.7	115.23		
Plants	4	11338.6	2834.65	62.97	0.0000
Concentrations	5	28952.9	5790.57	128.63	0.0000
Plants*concentrations	20	218.4	10.92	0.24	0.9996
Error	87	3916.6	45.02		
Total	119	44772.1			

Grand Mean 60.625 CV 5.07

Table S6: Mean percent (± SE) topical mortality of *C. maculatus* after 24 h exposure period treated with six different concentrations of plant crude-extracts of five plant species under laboratory conditions during 2018-2019.

**Source**	**DF**	**SS**	**MS**	**F**	**P**
Replications	3	460.00	153.333		
Concentrations	5	1210.00	242.000	6.81	0.0000
Plants	4	1488.33	372.083	10.48	0.0000
Plants *concentrations	20	231.67	11.583	0.33	0.9969
Error	87	3090.00	35.517		
Total	119	6480.00			

Grand Mean 9.0000 CV 6.22

Table S7: Mean percent (± SE) topical mortality of *C. maculatus* after 48 h exposure period treated with six different concentrations of plant crude-extracts of five plant species under laboratory conditions during 2018-2019.

**Source**	**DF**	**SS**	**MS**	**F**	**P**
Replications	3	49.17	16.389		
Concentrations	5	1164.17	232.833	5.29	0.0003
Plants	4	1136.67	284.167	6.46	0.0001
Plants*concentrations	20	123.33	6.167	0.14	1.0000
Error	87	3825.83	43.975		
Total	119	6299.17			

Grand Mean 14.083 CV 4.09

Table S8: Mean percent (± SE) topical mortality of *C. maculatus* after 72 h exposure period treated with six different concentrations of plant crude-extracts of five plant species under laboratory conditions during 2018-2019.

**Source**	**DF**	**SS**	**MS**	**F**	**P**
Replications	3	271.7	90.56		
Concentrations	5	8503.4	1700.67	23.39	0.0000
Plants	4	2067.7	516.93	7.11	0.0001
Plants*concentration	20	473.9	23.69	0.33	0.9969
Error	87	6326.8	72.72		
Total	119	17643.5			

Grand Mean 24.567 CV 3.71

Table S9: Mean percent (± SE) topical mortality of *C. maculatus* after 168 h exposure period treated with six different concentrations of plant crude-extracts of five plant species under laboratory conditions during 2018-2019.

**Source**	**DF**	**SS**	**MS**	**F**	**P**
Replications	3	184.3	61.42		
Concentration	5	27187.7	5437.53	49.91	0.0000
Plants	4	8847.8	2211.95	20.30	0.0000
Plants*concentration	20	1013.5	50.68	0.47	0.9729
Error	87	9478.2	108.95		
Total	119	46711.5			

Grand Mean 47.267 CV 14.00

Table S10: Mean percent (± SE) topical mortality of *C. maculatus* after 336 h exposure period treated with six different concentrations of plant crude-extracts of five plant species under laboratory conditions during 2018-2019.

**Source**	**DF**	**SS**	**MS**	**F**	**P**
Replications	3	578.2	192.72		
Concentrations	5	8966.3	1793.26	18.54	0.0000
Plants	4	13075.8	3268.95	33.80	0.0000
Plants*concentrations	20	965.5	48.28	0.50	0.9603
Error	87	8414.6	96.72		
Total	119	32000.3			

Grand Mean 79.925 CV 10.50
